# Reward Processing and Circuit Dysregulation in Posttraumatic Stress Disorder

**DOI:** 10.3389/fpsyt.2021.559401

**Published:** 2021-05-28

**Authors:** Yana Lokshina, Tetiana Nickelsen, Israel Liberzon

**Affiliations:** ^1^Department of Psychiatry and Behavioral Science, Texas A&M University Health Science Center, College Station, TX, United States; ^2^Texas A&M Institute for Neuroscience, Texas A&M University, College Station, TX, United States

**Keywords:** posttraumatic stress disorder, reward anticipation, reward outcome, anhedonia, context processing, emotion regualtion, nucleus accumbens, medial prefrontal cortex

## Abstract

Past decades have witnessed substantial progress in understanding of neurobiological mechanisms that contribute to generation of various PTSD symptoms, including intrusive memories, physiological arousal and avoidance of trauma reminders. However, the neurobiology of anhedonia and emotional numbing in PTSD, that have been conceptualized as reward processing deficits - reward wanting (anticipation of reward) and reward liking (satisfaction with reward outcome), respectively, remains largely unexplored. Empirical evidence on reward processing in PTSD is rather limited, and no studies have examined association of reward processing abnormalities and neurocircuitry-based models of PTSD pathophysiology. The manuscript briefly summarizes “state of the science” of both human reward processing, and of PTSD implicated neurocircuitry, as well as empirical evidence of reward processing deficits in PTSD. We then summarize current gaps in the literature and outline key future directions, further illustrating it by the example of two alternative explanations of PTSD pathophysiology potentially affecting reward processing via different neurobiological pathways. Studying reward processing in PTSD will not only advance the understanding of their link, but also could enhance current treatment approaches by specifically targeting anhedonia and emotional symptoms in PTSD patients.

## Introduction

Posttraumatic stress disorder (PTSD) is a highly debilitating psychiatric condition that produces immense suffering and incurs substantial individual and societal costs. It is often comorbid with other psychiatric disorders, among them, substance use (SUD) and depression. From the clinical perspective, PTSD is defined as a Trauma- and Stressor-Related Disorder with four clusters of symptoms that include intrusive trauma-related memories, avoidance of trauma reminders, physiological arousal, and negative mood and cognition *(DSM-5;* American Psychiatric Association, 2013). Importantly, the cluster of negative cognition/mood incorporate a broad range of symptoms, including (a) symptoms associated with inability to experience positive emotions (e.g., happiness, satisfaction, love) previously referred to as “emotional numbing” (b) symptoms of anhedonia i.e., loss of interest or motivation to participate in significant activities; and (c) symptoms that represent a generally negative cognitive-emotional state with exaggerated negative beliefs and distorted blame of self and others.

Over the past two decades, substantial body of research, had explored key neurobiological processes/circuits that could contribute to generation of PTSD symptoms like, intrusive memories, hyperarousal, or exaggerated physiological responses to trauma reminders (1–3), however, the neurobiology of negative cognition/mood symptoms in PTSD, remain largely unexplored. Importantly, as we describe in detail below, the fear learning mechanisms for example implicated in generation of other PTSD symptoms, do not readily explain generation of “emotional numbing” or anhedonia. One of the potential contributors to this is the fact that emotional numbing and anhedonia symptoms have not been clearly defined in prior PTSD research (4) and considered as conceptually similar, as both are characterized by diminished positive effect (5). However, emerging evidence suggests that emotional numbing and anhedonia might reflect related but separable processes in PTSD (with anhedonia focuses on anticipation and approach, while emotional numbing linked to the diminished capacity to experience pleasure), and, therefore, potentially generated by distinct neural circuits (6). Importantly, anhedonia has been linked to reward processing deficits in PTSD in prior studies (7), while association of emotional numbing to reward processing has been less clear (8, 9), although others linked emotional numbing to reward processing as well (6, 10).

Broadly speaking, reward processing has been conceptualized in psychological and neuroscience literature as the ability to feel pleasure when consuming or collecting the reward, as well as motivation to acquire it, and several components of reward processing have been identified, including reward wanting (or reward motivation), reward liking (or reward consumption associated with feeling of pleasure) and reward learning (11, 12). On the molecular level, these components of reward processing have been shown to rely on specific neurobiological mechanisms (7, 13), but molecular studies of reward processing in humans are still very limited, and in case of PTSD are almost non-existent, although, recently it has been suggested that anhedonia and emotional numbing in trauma-exposed individuals may be related to reward wanting and reward liking, accordingly (6). Thus while the nature of “negative cognition” symptoms suggest abnormal or diminished positive emotional response as well as low motivation to seek positive emotions, the literature empirically testing positive emotions (via manipulating reward empirically) in PTSD is quite limited and the results are not always consistent (14).

Furthermore, reward processing deficits have been also proposed to be the underlying mechanism of PTSD-major depression (MDD) and PTSD-substance use (SUD) comorbidities (15). Impaired reward processing has been consistently reported in MDD- and SUD-diagnosed patients, and MDD and SUD comorbidities are very frequent in PTSD patients (rates of 30–50% and 20–45% for PTSD-MDD and PTSD-SUD samples, respectively). Both comorbidities have also been marked by a more complicated clinical course with greater functional impairment and less favorable treatment results (16–19). Therefore, a better understanding of the underlying neurobiological mechanisms, including those involved in reward processing deficits, is urgently needed.

To date, empirical evidence of changes in reward processing in PTSD is both limited and equivocal, and no studies so far have examined the association of reward processing abnormalities and neurocircuitry-based models of PTSD pathophysiology, in spite of the emerging evidence implicating abnormal context processing in both (20, 21). Furthermore, existing neurocircuitry-based models of PTSD pathology do not specifically address reward processing deficits as well as the symptoms of anhedonia and emotional numbing in PTSD. Thus, a parsimonious explanation of PTSD pathology that incorporates reward processing deficits is still warranted. In the following paragraphs we briefly review the neurocircuits implicated in PTSD pathophysiology and symptoms development, the neurobiology of reward systems, and finally the emerging findings on reward processing in PTSD. We conclude by identifying the key gaps in the literature to date as well as outline direction for future research that will allow a more comprehensive understanding of PTSD neurobiology, inclusive of reward system related symptoms.

## Neurobiology of PTSD: Circuit Dysregulation

Over the past two decades, both studies using animal models (22–24) and functional neuroimaging research in humans have implicated PTSD-specific changes in neural circuits involving fear learning, memory and emotional processing (25, 26). More specifically, hyper- activation of insula, amygdala, and dorsal anterior cingulate cortex (dACC), as well as hypo- activation in ventral medial PFC (vmPFC) and impaired hippocampal function have been consistently reported (2, 27, 28). Furthermore, PTSD has been found to be associated with decreased functional connectivity within Default Mode Network (DMN, linked to self-referential processing and mind wandering with key nodes in PCC, vmPFC /sgPFC, and hippocampus), increased connectivity within Salience Network (SN, responsible for salience/threat detection and comprised of amygdala, insula and dACC), as well as with specific patterns of DMN-SN functional desegregation (26, 29–31). Alterations in relationships of DMN and SN with other networks such as Frontoparietal Network (FPN, involved in “top- down,” control emotional regulation and includes prefrontal cortical regions such as dlPFC), Dorsal Attention Network (DAN, involved in top-down voluntary orienting and comprised of MFG, posterior parietal lobe and frontal eyes fields) and Ventral Attention Network (VAN, linked to alerting and reorienting attention to unexpected stimuli and comprised of inferior frontal gyrus and temporal parietal junction regions) have been also reported. Specifically, increased FPN-SN connectivity (29) decreased DMN-FPN connectivity (32) as well as increased connectivity of DMN, SN with attention networks (33, 34) have been detected.

Our group had theorized that these neural circuits abnormalities subserve distinct brain functions, contributing to different aspects of PTSD symptomatology, like fear learning (FL), threat detection (TD), executive function/emotion regulation (EF/ER), and context-processing neurocircuits (CP) (20).

### Fear Learning (FL) Circuitry

This circuitry enables fear acquisition, fear generalization and fear extinction processes, and altered fear extinction and fear overgeneralization have been repeatedly reported in PTSD (35, 36). These processes involve the basolateral complex of the amygdala (BLC), where fear learning takes place. Amygdala and BLC subnetworks are interconnected with the vmPFC and the hippocampus, that work in conjunction to regulate fear/extinction learning. Indeed, impaired extinction recall in PTSD is manifested in hyperactivation of amygdala and hypoactivation of mPFC, raising the possibility that the fear learning deficit in PTSD might stem from extra amygdala regulatory regions (20).

### Threat Detection (TD) Neurocircuitry

It controls a more general ability to detect and orient to salient cues in the environment, whether positive or negative, and alterations in threat detection could provoke hypervigilance and bias toward trauma cues. Salience or threat detection is subserved by the salience network (SN) regions involving anterior ACC, insula/operculum and amygdala. PTSD patients indeed exhibit hypervigilance and study liked PTSD with attentional bias toward threat and, consequently, exaggerated threat detection (26), manifested in heightened responsivity of insula, amygdala, dACC and increased connectivity within SN (37).

### Emotional Regulation/Executive Function (EF/ER) Neurocircuitry

A different cognitive emotional function that had been linked to PTSD pathophysiology is emotional regulation/executive function (EF/ER). EF/ER neurocircuitry regulates one's ability to modulate emotions by engaging higher cognitive/executive functions, such as redirecting attention away from emotional stimuli, engaging cognitive reappraisal etc. It involves prefrontal cortical regions, including dorsolateral, ventrolateral, lateral orbitofrontal, dorsomedial PFC as well as dACC. PTSD has been characterized by exaggerated emotional responses, diminished ability to redirect attention from emotional stimuli, and diminished capacity for reappraisal or suppression of emotions (38, 39). Decreased activation of dlPFC and dmPFC, as well as decreased connectivity between DMN and FPN, in contrast to increased connectivity of DMN with attention networks, have been detected in PTSD (40, 41).

### Context Processing (CP) Circuitry

Finally, more recently we have proposed that context processing abnormalities may serve as a parsimonious model explaining various PTSD symptoms/deficits (20). Context processing (CP) circuitry supports representation and retrieval of general or “background” information in the environment, and this circuitry is critical to modulate responses to various but especially ambiguous cues to best fit the current situation. This circuitry is dependent on pattern completion and pattern separation functions performed by hippocampus, and its interconnection with mPFC and thalamic nuclei, such as nucleus reunions. PTSD have been associated with context processing deficits, and it has been suggested that impairments in pattern separation and pattern completion processes could lead to dominance of fear over safety memories, inappropriate cue-related behavioral responsivity, and hypervigilance. Hypoactivation of hippocampus and vmPFC and reduced connectivity between these regions have been indeed reported in PTSD (42).

Interestingly, while deficits within CP circuitry have been implicated in a core PTSD pathology (43), it has been primarily investigated in the situations of fear, threat and danger. However, patients with PTSD demonstrate difficulties in understanding contextual nuances in various situations, including the non-threating one; for example when reward is expected (44), and context processing had been identified as one of the key aspects in addiction ((45)), and as we had mentioned addiction is highly prevalent in PTSD patients. PTSD has been associated with altered expectancy and lower satisfaction with reward (7, 14), and changes in activation of the regions associated with reward processing, including nucleus accumbens (NAc) have been reported in PTSD, raising the question regarding the role context processing deficits in reward processing abnormalities in PTSD and in PTSD/SUD comorbidity. In the next paragraph, we will describe the neurobiology of reward processing, and then provide evidence of PTSD-related alterations in it.

## Neurobiology of Reward Processing

### Animal Studies

Reward processing is a multi-faceted construct, and three distinct components of reward functioning have been proposed, including reward wanting (or reward motivation), reward liking (or reward consumption) and reward learning (11, 12). Reward wanting is defined as anticipatory motivation toward obtaining a reward, reward liking is linked to experience of pleasure associated with receiving a reward, while reward learning refers to learning about cues and actions associated with reward, as well as to adaptation of one's behavior based on this knowledge (11). Decades of animal research have provided a rich account of the neurobiological mechanisms underlying these three components (46–48).

#### Reward Wanting

This component has been consistently found to be controlled by mesolimbic and mesocortical dopamine (DA) pathways. More specifically, DA neurons in ventral tegmental area (VTA) have been shown to target nucleus accumbens (NAc), amygdala, and hippocampus in the mesolimbic pathway and cortical regions including mesial (MFC) and orbitofrontal cortices (OFC), in the mesocortical pathway (49, 50). Importantly, evidence from animal studies have indicated that when dopamine is reduced, reward motivation is impaired (51, 52). In addition, glutamate afferents convey signals to the NAc about motivational stimuli from prefrontal cortex, basolateral amygdala, and ventral hippocampus (53). Additionally, the NAc receives excitatory afferents from the thalamus and projects back via ventral pallidum (VP) to midline and intralaminar thalamic nuclei that projects to prefrontal cortex, thus completing cortico–striato–pallidal–thalamic loops/cortical-basal ganglia circuit (46, 54, 55).

#### Reward Liking

**Figure 1:**
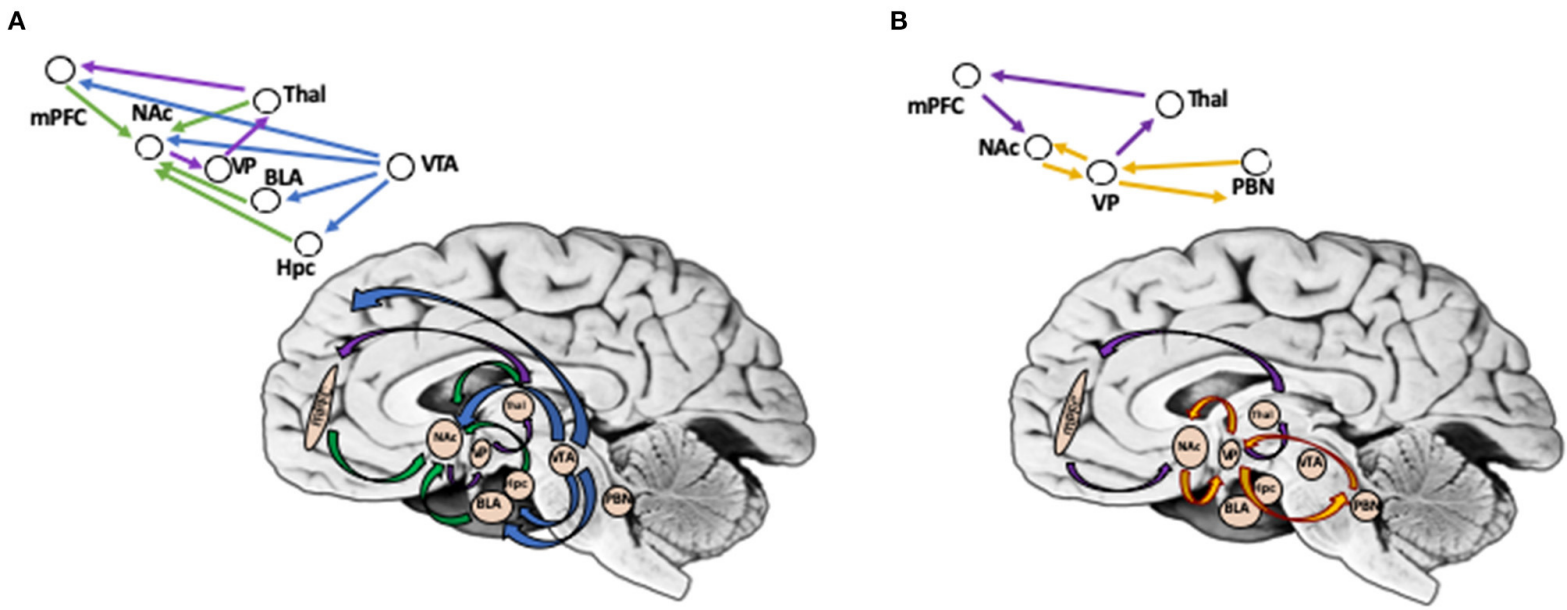
**(A)** Brain Circuits Involved in Reward Wanting (Anticipation). Reward wanting include mesolimbic and mesocortical dopamine pathways (shown in blue). DA neurons in ventral tegmental area (VTA) target nucleus accumbens (Nac), basolateral amygdala, and hippocampus in mesolimbic pathway, and cortical regions (mPFC) in mesocortical pathway. In addition, glutamate afferents convey signals to the NAc from mPFC, basolateral amygdala, hippocampus, and thalamus (shown in green). NAc projects via ventral pallidum (VP), to thalamic nuclei, that projects to prefrontal cortex, thus completing cortico-striato-pallidal-thalamic loops (shown in purple). **(B)** Brain Circuits Involved in Reward Liking (Outcome). Reward liking link together “opiod hotspots,” one of them being in the nucleus accumbens (Nac) shell, while another in posterior part of ventral pallidum (VP) (shown in yellow). It has been proposed that liking may be sub-served by a hierarchical circuit that in addition to NAc and VP includes areas in the forebrain, such as parabrachial nucleus, as well as in brainstem. Cortico-striato-pallidal-thalamic loops include projections from mPFC to NAc; NAc in turn projects via VP, to thalamic nuclei, which then targets mPFC (shown in purple). PFC, prefrontal cortex; NAc, nucleus accumbens; VP, ventral pallidum; VTA, ventral tegmental area; BLA, basolateral amygdala; Hpc, hippocampus; Thal, thalamus; PBN, parabrachial nucleus. *Background photo courtesy of Dr. Leonard E. White.

In contrast to reward wanting, reward liking is primarily mediated not by DA projections but by the opioid system that is responsible for generation of pleasurable reactions at specific sites in limbic structures, or “opioid hotspots,” one of them being in the medial shell of NAc, while another in the posterior part of the ventral pallidum. In addition to the opioid system, endocannabinoids have been shown to enhance “liking” reactions in a NAc hotspot that overlaps the mu opioid site (48, 56). It has been proposed that hedonic hotspots may be functionally linked together into an integrated hierarchical circuit that combines multiple areas in the forebrain, such as parabrachial nucleus, as well as in the brainstem (47, 57). Figure 1 illustrates brain circuits involved in reward wanting and liking.

#### Reward Learning

It has been shown to be encoded by fast phasic DA bursts in VTA that activate DA receptors in NAc, in a circuit with prefrontal cortex (in contrast to reward wanting, where DA acts not on fast, but on the intermediate time scale). When outcome is better than expected (positive reward prediction error), D1 receptors, sensitive to higher DA concentrations in NAcc are activated, whereas when an unexpected reduction of reward occurs (negative reward prediction error), D2 receptors are activated which lead to depressed activity of DA neurons. In addition to VTA-NAc projections, the afferents from lateral habenula to VTA and mesopontine rostromedial tegmental nucleus (RMTg) have been found to be implicated in reward learning, and specifically in negative reward prediction error (58–60).

### Human Studies

In human research however, most of the studies have focused on two components of reward processing, reward anticipation (or anticipatory phase) and reward outcome (or consummatory phase), roughly corresponding to reward wanting and reward liking in animal studies (61–63). It is important to note here that reward outcome may incorporate a broader range of processes than reward liking alone, as not only emotional/affective components included, but also cognitive appraisal of reward delivery. In addition, both reward anticipation and reward outcome may incorporate aspects of reward learning, when expectation of the reward has been shaped, and individual compares predicted and actual gains or losses.

In general, empirical findings regarding neurobiological mechanisms underlying aforementioned components of reward processing are generally consistent with animal research.

#### Reward Anticipation and Reward Outcome Activation Patterns

In some of the earlier studies (64), reported similar patterns of activation between two phases of reward processing. Activations in nucleus accumbens (NAc), sublenticular extended amygdala (SLEA), and thalamus have been reported during both the anticipation and reward outcome phases, while activation in orbitofrontal cortex (OFC) has been revealed only during the reward outcome phase. Interestingly activations of the NAc, SLEA, and hypothalamus have been shown to increase in response to high monetary gains in the reward outcome phase, as well as to higher expected value of monetary gains in the anticipation phase.

However, others (65, 66) have reported differential activation during two phases of reward processing, and this has been replicated in recent studies and by different research groups (63, 67, 68).

#### Reward Anticipation

Knutson et al. (66) have reported that NAc is primarily recruited by anticipation of monetary reward, and when anticipated rewards are not obtained (reward prediction error), NAc is suppressed In addition to NAc, medial caudate and medial prefrontal cortex (mPFC) have been activated during anticipation of reward. Interestingly, the activations observed in NAc and mPFC that were proportional to the magnitude of anticipated reward have been replicated in further studies (67, 68), and the mPFC activation has also traced anticipated probability of rewards (69, 70). On the other hand, activations in medial caudate and thalamus traced not only the magnitude of the reward, but also of anticipated punishment. A meta-analysis of 20 fMRI studies suggested that the NAc responds robustly during anticipation of monetary gains, but not during the outcome phase (71), and it has been replicated in recent studies (63). Furthermore, activations in NAc have been linked to reward prediction error, with decreased activity of NAc being detected when rewards are omitted or smaller than expected (72).

#### Reward Outcome

For the reward outcome, ventromedial PFC (vmPFC) has been activated, while omission of anticipated rewards has suppressed vmPFC activity (63, 65, 73). Additionally, dorsal caudate and parietal cortex have been recruited in reward outcome condition.

### Animal and Human Research: Conceptual and Methodological Disparities

What could be the source of these differences in findings between human and animal research?

#### Understanding at the Molecular Level

First, some of the differences in underlying components of reward processing might be difficult to detect in human research, as these are described on a molecular level (such as involvement of opioid system in nucleus accumbens (NAc) for reward liking, and dopamine (DA) afferents to NAc in reward wanting), while human reward studies on a molecular level are yet to be performed.

#### Regions of Interest (ROI) Used

Second, without invasive approaches it is very hard to target small regions, such as NAc core and shell, or lateral habenula. Thus, while animal studies could target directly NAc core and shell for wanting and liking components, respectively, human studies sometimes examined a larger ventral striatum region that includes not only NAc, but also olfactory tubercle, and sometimes rostroventral putamen and the medial caudate nucleus (62, 63, 74).

#### Type of Reward Used

Lastly, in contrast to animal studies that used food for reward (or drugs in addiction models), the aforementioned human research has primarily focused on abstract reward, such as monetary gains. Some human studies incorporated pleasant pictures (75–77), or happy faces (72, 78, 79) as positive rewarding stimuli, and in these studies, activations in the ventral striatum have been reported during the reward outcome phase.

Monetary gains, pleasant pictures, and happy faces – rewards most often used in human studies have been all conceptualized as abstract or secondary rewards, in contrast to pleasant tastes, smells, touch, sounds, and sights often considered as the primary rewards (63, 79–82). Haber and Knutson (62) have hypothesized that for primary rewards, the anticipation recruits ventral striatum and orbitofrontal cortex (OFC), while the reward itself elicits only OFC, and that primary rewards tend to activate more posterior OFC regions, while abstract rewards - more anterior OFC regions (62). Sescousse et al. (83) have also shown that monetary gains tend to engage the most anterior portion of the OFC, while primary rewards, food, have been represented in the anterior insula. Clearly, more empirical support is needed for these hypotheses. One can also wonder whether greater involvement of cortical regions, like the mPFC, in human research is also facilitated by (a) the abstract character of rewards used in human studies and (b) the engagement of cognitive appraisal of reward outcomes in human subjects.

In summary, both animal and human research have indicated the importance of cortical- basal ganglia circuits in reward processing, with an established role of NAc in anticipation of reward, and less clearly defined roles for cortical regions (like OFC and mPFC) during both anticipation and reward outcome phases in human studies. Furthermore, animal and human studies have shown that OFC encodes the value of available outcomes (84–86). Importantly, it has been demonstrated that the medial OFC maintains a representation of the expected reward associated with particular cues (87–89). Clearly, for both animal and especially human research, additional work will be required to better understand the specific roles of these regions in responses to different type of rewards, in different species, both on the molecular and the brain circuits levels.

## Reward Processing in PTSD

Limited prior research has suggested potential alterations in reward anticipation and outcome components of reward processing, with PTSD patients exhibiting both lower expectancy and lower satisfaction with reward, as evident from less effort expanded to view positive rewarding stimuli (7, 10). However, only a few studies have examined neural mechanisms associated with these two components in PTSD. Furthermore, while some agreement can be found among studies of the reward outcome phase, evidence regarding reward anticipation is inconclusive at best.

### Reward Outcome

Several studies have indicated that PTSD patients, compared to trauma-exposed controls, exhibit decreased ventral striatal and decreased medial prefrontal cortex (mPFC) activation to reward (relative to loss) (10, 90). Admon et al. (91) have reported that PTSD-related decrease in striatal responses to reward is not present prior to deployment, i.e., before trauma exposure and PTSD development. Importantly, same striatal responses to reward (relative to loss) have been linked to anhedonic PTSD symptoms severity (10). It is noteworthy that in these studies, reward condition has been compared to loss (punishment condition), therefore, the task included not only positive, but also negative reinforcement, potentially engaging different/additional mechanisms. When the response to positive/rewarding stimuli has been compared to the neural one, no differences in ventral striatum activations have been detected among the groups, however decreased dmPFC responses to positive stimuli have been detected in PTSD patients. In concert, in study by (92) reduced dmPFC activity in response to a positive social audio-script (presumably rewarding) has been related to higher severity of emotional numbing or “inability to experience positive emotions” in PTSD. In addition, increased insula responses have been related to decreased positive affect in response to rewarding stimuli in PTSD patients (92).

### Reward Anticipation

The existing evidence regarding reward anticipation component brain circuitry, as already stated, is very limited and rather contradictory. Elman et al. (10) reported no differences in neural activation patterns during expectation of reward, however altered response of NAc has been observed during the expectation of an aversive outcome, which is at odds with the results of meta-analysis mentioned earlier (71). Furthermore, a recent study in PTSD patients reported higher activation in the NAc, putamen, and amygdala during anticipatory phase (14), which is counterintuitive, if the diminished reward motivation in PTSD is indeed present. Interestingly, a recent resting-state study, that compared patterns of functional connectivity in PTSD and comorbid PTSD-major depression (MDD) reported that patients with comorbid PTSD and depression, exhibited decreased connectivity in striatal-subcortical pathways, such as nucleus accumbens (NAc)-thalamus, and NAcc-hippocampus, as well as decreased connectivity between basolateral amygdala and orbitofrontal cortex, all correlated with the severity of depressive symptoms (93). In this study, however, distinct clusters of PTSD symptoms (including negative mood and cognition) have not been specifically studied.

In sum, the empirical findings on PTSD-related changes in neurobiological mechanisms of reward anticipation and outcome are rather limited. Decreased mPFC activation during the outcome phase in PTSD patients has been reported across number of the studies (91, 94) however, diminished activations of ventral striatum (NAc presumably) have been reported in some studies (10), but not others (95). Furthermore, the question of PTSD-related changes during reward anticipation remains wide open, as one study reported increased activation of NAc in PTSD patients, while others showed no changes in NAc (10). Clearly additional studies are urgently needed to: (a) determine whether and what kind of reward processing abnormalities are indeed present in PTSD, and (b) are they linked to the mechanisms generating “negative cognition” or contributing to PTSD/SUD and PTSD/MDD comorbidity. Finally, additional work will be needed to establish the links between PTSD related pathophysiology described in previous paragraph to neurobiological alterations underlying reward processing in PTSD.

## Gaps and Future Studies

So, what are the key areas that need to be addressed to answer the question whether reward processing deficits are involved in anhedonia and emotional numbing symptoms (specifically, included in the negative mood and cognition symptoms cluster) in PTSD?

### Delineating Neural Mechanisms of Distinct Components of Reward Processing

First, while animal research has outlined three components of reward processing (reward wanting, reward liking, and reward learning), and delineated specific brain regions and neural mechanisms that subserve these functions, the homology to human research so far is largely limited to the reward anticipation phase.

### Understanding the Reward Outcome Phase

With respect to reward “liking” the participation of nucleus accumbens (NAc) has to be further confirmed, but importantly the limited human research raises the possibility that the reward outcome phase might incorporate a broader range of processes, not limited to hedonic reactions, including cognitive evaluation of the received rewards, and thus engaging additional cortical regions. This could explain the involvement of cortical regions like mesial (MFC) and orbitofrontal cortices (OFC) in some human studies.

### Type of Reward Used

It is also possible that different types of rewards trigger different hedonic reactions. In animal studies the primary reward – commonly food, has been investigated, while in humans, the major focus has been devoted to the abstract rewards, such as money gains or pleasant pictures. It is possible that the abstract nature of the reward engages cortical regions. However, it has also been suggested that different parts of OFC may be implicated in primary vs. abstract rewards. Thus, specific roles of OFC, mPFC and other regions, in reward processing in general and in human reward outcome in particular, have to be further delineated.

### Understanding at the Molecular Level

Furthermore, research on reward processing in humans have primarily (and almost exclusively) focused on circuit level dysregulation, which presents a limited perspective, as distinct neurotransmitters and modulatory systems within the same regions (NAc) may be important for different components of reward processing, such as opioid system for reward liking, and DA for reward wanting. Future research should examine human reward processing on molecular level within the anatomical context.

### Regions of Interest (ROI) Used

Furthermore, some of the human research and especially research that involves patients groups like PTSD, is not consistent with respect to the choice of regions of interest (ROI). While some studies have examined involvement of NAc (in line with animal studies), others reported results using a broader - “ventral striatum” ROI. Furthermore, few studies have discussed the involvement of regions like dorsal striatum, dorsal putamen and caudate, that are more likely to be involved in goal-directed behavior rather than reward wanting or liking.

With respect to reward processing abnormalities in PTSD, while the nature of “negative cognition” symptoms, the high comorbidity with major depression (MDD) and substance use (SUD), and initial studies of reward outcome all point out to possible abnormalities of reward processing circuitry, much work is required to clearly establish this fact. As stated above, the evidence is limited and on the circuit level, it is not clear what exact brain regions are implicated in PTSD-related changes in “reward wanting.” Furthermore, it is not fully confirmed, if the reward outcome response in PTSD is diminished, what exact circuits are involved and how these changes are related to “negative cognition” or comorbidities that triggered these questions.

Additionally, activity of reward circuitry may be affected by inflammation and childhood trauma, which both constitute the risk factors for PTSD. More specifically, PTSD has been consistently associated with elevated levels of pro-inflammatory markers like C reactive protein (CRP) and pro-inflammatory plasma cytokines, including interleukins (IL)-1 and IL-6 (96, 97), and recent studies have demonstrated that activity of ventral striatum in response to reward is negatively correlated with circulating concentrations of IL-6 and CRP (98). Moreover, increased CRP has been found to be associated with decreased connectivity between ventral striatum and vmPFC (99, 100). As for the childhood trauma, it has been linked to decreased connectivity between NAc and OFC (101), and reduced activity of ventral striatum in response to reward ((102)). Future studies will be needed to examine the effects of inflammation and childhood trauma on reward circuitry in PTSD.

### Circuit Dysregulation and Reward Processing Deficits in PTSD: Possible Explanations of PTSD Pathology

**Figure 2:**
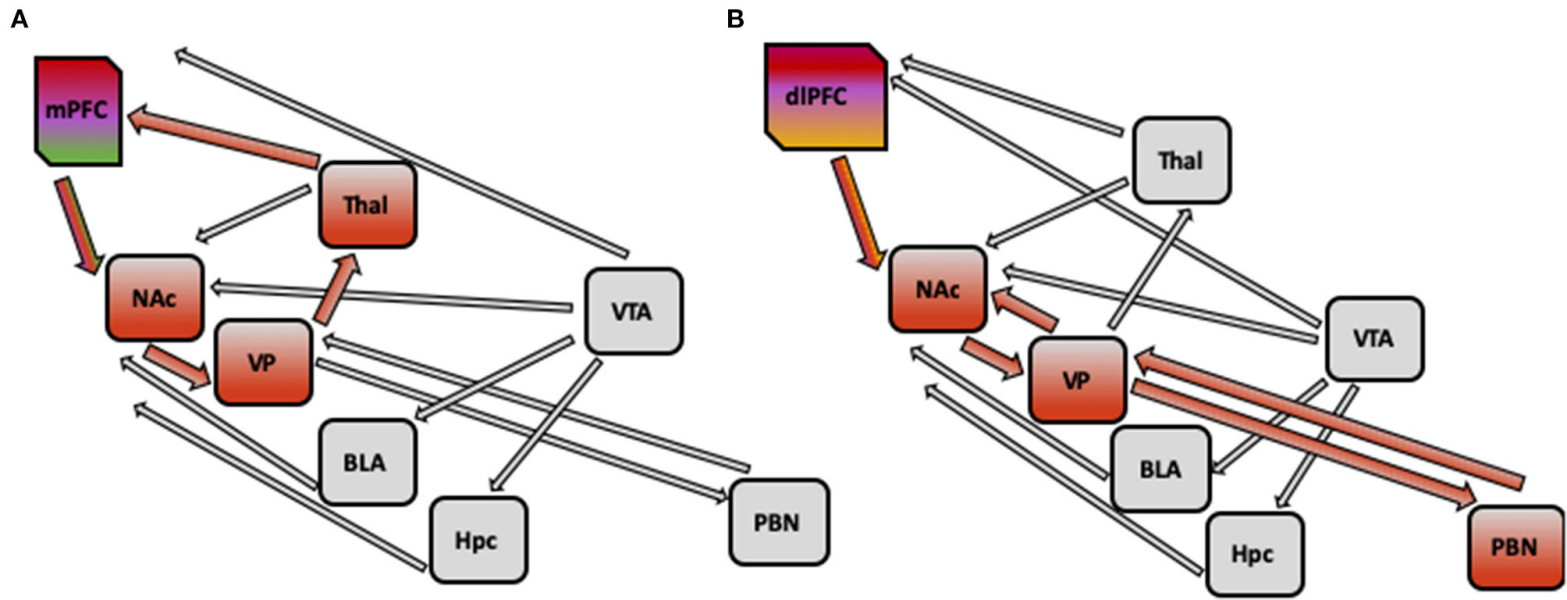
**(A)** Hypothetical pathway implicated in PTSD pathology: impaired context processing linked to alterations in reward processing. Impaired context processing reflected in diminished activity of medial PFC may affect the reward anticipation via altering the activity of glutamate afferents from medial PFC to nucleus accumbens (NAc), which may lead to decreased activation of NAc. Furthermore, as Nac projects to ventral pallidum, which in turn projects to prefrontal cortex via thalamic nuclei, the activity of this cortical loop may be changed. **(B)** Hypothetical pathway implicated in PTSD Pathology: impaired emotion regulation linked to alterations in reward processing alterations in emotion regulation (such as inability to switch attention from trauma-related cues, or diminished cognitive appraisal) reflected in decreased activation of dorsolateral PFC and dorsomedial PFC may lead to diminished positive affect with altered activity of opioid system in nucleus accumbens (NAc) and ventral pallidum (VP). Decreased activity of NAc and VP may be present, and weaker connections with areas in forebrain, such as parabrachial nucleus (PBN), could be detected. mPFC, medial prefrontal cortex; NAc, nucleus accumbens; VP, ventral pallidum; VTA, ventral tegmental area; BLA, basolateral amygdala; Hpc, hippocampus; Thal, thalamus; PBN, parabrachial nucleus.

Finally, existing models of PTSD pathology, such as fear learning, threat detection, emotion regulation and context processing models, do not directly describe regions associated with reward function, such as nucleus accumbens (NAc). It has been hypothesized that context processing deficits as seen in PTSD in diminished mPFC activity, may affect the reward processing, specifically, reward wanting or anticipation via altering activity of glutamate afferents from PFC to NAc. If reward anticipation is indeed impaired in PTSD and it is reflected in altered mPFC input to NAc, in turn affecting its projection to VP, which in turn projects to PFC via thalamic nuclei, changing activity of cortical loop. Figure 2A illustrates hypothetical pathway linking context processing and reward anticipation in PTSD. However, additional empirical data is needed to confirm this link and to further our understanding of anhedonia and negative cognitions symptoms in PTSD.

It is also possible, alternatively, that the inability to switch attention from trauma-related or aversive cues, or diminished cognitive appraisal may lead to diminished positive affect (related to emotional numbing and reflected in activity of opioid system in NAc and ventral pallidum) in PTSD patients. Thus, alterations in emotion regulation circuitry (including decreased activation of dlPFC and dmPFC, as well as decreased connectivity between DMN and FPN) in PTSD can give rise to impairment in reward processing by deficient DLPFC input to NAc, resulting in altered signaling to VP, and weakened connectivity with forebrain, and specifically parabrachial nucleus. Figure 2B illustrates hypothetical pathway linking emotion regulation and reward liking or outcome in PTSD. Future empirical studies will be needed to distinguish between these competing explanations.

Understanding of the neurobiology of reward processing alterations in PTSD, as well as its possible associations with the existing models of PTSD pathology are important not only to our knowledge of symptom formation, but it has the potential to enhance current treatment approaches and specifically target anhedonia and emotional symptoms in PTSD patients. Furthermore, as reward processing deficits has been proposed to be the mechanism underlying PTSD-MDD and PTSD-SUD comorbidities, and both of these comorbidities have been marked by greater functional impairment and worse treatment results, understanding of the neurobiological mechanisms involved in reward processing deficits might be central to the targeted approach to treatment of these comorbidities.

## Data Availability Statement

The original contributions presented in the study are included in the article/Supplementary Material, further inquiries can be directed to the corresponding authors.

## Author Contributions

YL, TN, and IL: contributed to the writing of the manuscript. YL and IL: developed the theoretical formalism. IL: provided critical feedback and supervised the project. All authors contributed to the article and approved the submitted version.

## Conflict of Interest

The authors declare that the research was conducted in the absence of any commercial or financial relationships that could be construed as a potential conflict of interest.
