# Supplementary material for: Reward Processing and Circuit Dysregulation in Posttraumatic Stress Disorder
Source: Front Psychiatry. 2021 May 28;12:559401. doi: 10.3389/fpsyt.2021.559401 (PMC8193060; doi:10.3389/fpsyt.2021.559401)
Supplement: Supplementary file 1 [file Data_Sheet_1.PDF]

Reward Processing in PTSD

Study	Reward Processing Component	Type of Reward	Negative Reinforcement Included	Results: PTSD vs Controls
Elman et al. (2009)	Reward Anticipation & Reward Outcome	Monetary gains	Included. Reward condition compared to loss (Punishment)	• Reward Anticipation: No between-group differences for anticipation of reward<br>• Reward Outcome: PTSD group exhibited decreased bilateral (1)ventral and (2)dorsal striatal activations, compared to controls
Frewen et al. (2010), (2012), (2013)	Reward Outcome	Imagery of positive social scripts	Included. Positive social scripts were compared to negative social scripts	• Reward Outcome: PTSD group exhibited (1)reduced dmPFC and (2)increased insula activations, compared to controls
Aldmon et al. (2013)	Reward Outcome	Matching chips (Domino game)	Included. Nonmatching chips were associated with punishment if uncovered	• Reward Anticipation: No between-group differences for anticipation of reward<br>• Reward Outcome: Increased NAc activation in response to reward (correlated with PTSD symptoms)
Killgore et al. (2013), (2018)	Reward Outcome	Imagery of happy faces	Not included. Compared to neutral faces	• Reward Outcome: PTSD group exhibited increased amygdala activity, compared to controls

Study	Reward Processing Component	Type of Reward	Negative Reinforcement Included	Results:<br>PTSD vs Controls
Boukezzi et al. (2020)	Reward Anticipation & Reward Outcome	Monetary gains	Included. Reward condition compared to loss (Punishment)	• <u>Reward Anticipation:</u> PTSD group exhibited increased (1)nucleus accumbens, (2) amygdala, and (3)putamen, compared to controls<br>• <u>Reward Outcome:</u> PTSD group showed a unique significant activation in the caudate nucleus
